# A systematic review on the associations between low back pain and frailty in community-dwelling older adults

**DOI:** 10.1186/s12998-025-00601-3

**Published:** 2025-09-29

**Authors:** Munkh-Erdene Bayartai, Sum Yi Lam, Kei Yan Chan, Wai Ying Lau, Suet Ying Lee, Chun Yin Yip, Jeremy R. Chang, Edmond C. M. Wong, Manuela L. Ferreira, Arnold Y. L. Wong

**Affiliations:** 1 Department of Physical and Occupational Therapy, School of Nursing, Mongolian National University of Medical Sciences, Ulaanbaatar, Mongolia; 2 Department of Rehabilitation Sciences, The Hong Kong Polytechnic University, Kowloon, Hong Kong SAR China; 3 The George Institute for Global Health, University of New South Wales, Sydney, NSW Australia; 4 Research Institute for Smart Ageing, The Hong Kong Polytechnic University, Kowloon, Hong Kong SAR China

**Keywords:** Frailty, Low back pain, Older adults, Aging, Systematic review, Correlation

## Abstract

**Background:**

Frailty and low back pain (LBP) may negatively affect each other in older adults, yet no systematic review has summarized their cross-sectional, temporal, or causal associations. Exploring these associations could provide valuable insights for reducing frailty risk in older adults with LBP. This systematic review aimed to consolidate evidence on the association between frailty and LBP in older adults.

**Methods:**

Four databases (EMBASE, CINHAL, MEDLINE, and SPORTDiscus) were systematically searched from their inception until July 31, 2024. Studies investigating the association between LBP, regardless of chronicity, or LBP-related disability and frailty in older adults were included. LBP was defined as pain occurring between the 12th ribs and inferior gluteal folds. Due to the lack of consensus on the definitions of older adults or frailty, studies were included based on the authors’ definitions. Three pairs of independent reviewers screened abstracts and full texts, extracted data, assessed risk of bias, and determined the certainty of evidence.

**Results:**

Out of 1,690 articles identified, six cross-sectional studies and one prospective study were included. Low-certainty evidence from four cross-sectional studies suggested that both acute and chronic LBP, with odds ratios from 1.34 to 7.50, separately showed significant correlation with frailty. Pre-frail or frail older adults reported higher chronic LBP intensity, scoring 0.5 to 0.8 points more on the numeric rating scale, and greater LBP-related disability, with scores 1.7 to 7.2 points higher on the Roland Morris Disability Questionnaire, compared to non-frail counterparts. However, there was low-certainty evidence that acute LBP intensity was unrelated to frailty. Very low-certainty evidence from the prospective study indicated that higher acute LBP intensity and disability were associated with transitioning from non-frail to prefrail or frail status.

**Conclusions:**

Our systematic review revealed that older adults with higher LBP intensity or associated disability were more likely to have prefrail or frail status, albeit with low-certainty evidence. However, the findings are limited by the small number of studies, especially prospective research. Future high-quality research should clarify the causation between LBP intensity or disability and frailty in community-dwelling older adults. Research should also explore potential mediators or moderators influencing the LBP-frailty association. These findings could help develop effective prevention and rehabilitation strategies to mitigate the impacts of LBP on frailty, or vice versa.

## Introduction

By 2050, approximately 25% of the global population is expected to be aged 65 years or older [1], leading to an increase in age-related health issues. Frailty is particularly common among these concerns. Over half of community-dwelling older adults are susceptible to frailty, with a higher incidence observed in women than men [2]. Frailty is a complex condition influenced by multiple factors that increase older adults’ vulnerability to adverse health outcomes, primarily due to reduced physiological reserves [3] and decreased ability to cope with stressors [4]. This condition is associated with reduced mobility, increased risks of falls, disabilities, hospitalization, and mortality [5–7]. Additionally, frailty negatively impacts the quality of life for older adults and significantly raises healthcare costs [8].

Chronic musculoskeletal pain is another prevalent geriatric health issue, affecting 66% of those aged 65 and older [9]. Persistent pain in this population is thought to contribute to diminished physiological reserves and impaired mobility, thereby increasing the likelihood of developing frailty [10, 11]. A systematic review of prospective longitudinal studies revealed that individuals with multi-site chronic pain have double the risk of developing frailty within 12 months, suggesting a potential causal relationship between chronic pain and the onset of frailty [12]. However, the certainty of evidence regarding whether this causal relationship applies to all types of chronic musculoskeletal pain remains unclear.

Among various types of musculoskeletal pain, low back pain (LBP) is the most prevalent, affecting 33% of older adults globally. It is projected that the global prevalence of LBP will surpass 800 million people by 2050 [13]. Chronic LBP, lasting three months or more, can lead to numerous adverse effects, including psychological disorders (e.g., depression), fear avoidance behaviors, withdrawal from social and recreational activities, accelerated cognitive decline, general deconditioning, and physical inactivity [14–16]. These detrimental effects can significantly decrease the quality of life for older adults [17, 18] and increase their risk of frailty or falls [19].

Given the high prevalence of frailty and LBP among the aging population, several cross-sectional studies have identified a potential association between acute or chronic LBP and frailty status, along with the physical and psychological deterioration associated with frailty [20–22]. Furthermore, a prospective study revealed that older adults with LBP, initially classified as non-frail, were more likely to transition to pre-frail and frail states after one year. These results highlight the vulnerability of older adults with LBP to the gradual development of frailty syndrome over time [23]. However, as the cross-sectional and temporal associations between LBP and frailty are relatively new research areas, a comprehensive search and synthesis of evidence are needed to determine the certainty of these associations.

The current systematic review aimed to summarize the evidence regarding the cross-sectional, temporal, or causal associations between LBP and frailty. Given that LBP may increase the risk of frailty, the findings could provide valuable insights for researchers and clinicians, including geriatricians and physiotherapists, in understanding the association, and developing specific prevention or intervention strategies to reduce the risk of frailty in older adults with LBP.

## Methods

The review protocol was registered on the PROSPERO (CRD42023442058). This review was reporting according to the Preferred Reporting Items for Systematic Reviews and Meta-Analyses (PRISMA) statement [24].

### Literature search strategy

MEDLINE ALL (via OVID), EMBASE (vias OVID), CINAHL (via EBSCOhost), and SPORTDiscus were systematically searched from their inception to July 31, 2024. The search strategies used a combination of keywords and medical subject headings related to LBP, frailty, and older adults. An experienced researcher with a decade of experience in conducting systematic reviews developed the search strategies. Details of the search strategies are provided in Appendix I. The reference lists of the included studies and forward citation tracking via Scopus were screened to identify additional relevant articles. Additionally, the corresponding authors of the included studies were contacted via emails to uncover additional pertinent publications.

### Selection criteria

Studies were eligible for inclusion if they met the following criteria:

1. Design: cross-sectional, case-controlled, prospective, or retrospective studies examining the association between LBP-related clinical outcomes and frailty in community-dwelling older adults.
2. Population: older adults with or without LBP of any chronicity, and with or without frailty for comparison. LBP was defined as pain between the last ribs and the inferior gluteal fold [25]. Definitions of older adults varied across studies. Therefore, authors’ definitions were accepted to encompass all potential relationships. Frailty should be clearly defined based on criteria related to weight loss, muscle strength, fatigue, ambulation, inactivity, and/or comorbidities [6, 26, 27].
3. Measurement: LBP intensity or LBP-related disability quantified using validated scales, such as the Numeric Rating Scale (NRS) for pain and the Oswestry Disability Index (ODI) for LBP-related disability. Frailty determined using the Cardiovascular Health Study (CHS) criteria, the FRAIL scale or modified frailty index. For example, pre-frailty is defined as the presence of 1 or 2 out of 5 frailty phenotypes in CHS: (1) shrinking; (2) weakness; (3) exhaustion; (4) slow gait speed; and (5) low-energy expenditure [6]. Individuals with three or more positive phenotypes are classified as frail [6].
4. Temporal association or prediction: Studies should provide follow-up timepoints to estimate such associations.

Studies were excluded if they: (1) were dissertations, abstracts, editorials/letters, case reports, or conference proceedings; (2) included older adults with specific back pathologies (e.g., cancer/tumor, spinal infection, or fracture); or (3) did not report statistical findings or raw data, including association measures (e.g., ORs, Pearson or Spearman correlations) and the corresponding 95% confidence intervals, even after attempts to obtain such information by contacting the corresponding authors.

### Selection of studies

Six reviewers (M.B., S.L., V.L., P.Y., Y.C and H.L.) were organized into three pairs to independently screen the titles and abstracts of identified citations. The first 100 citations served as a pilot to evaluate inter-reviewer agreement using Cohen’s kappa coefficient. Disagreements within pairs were resolved through discussion, and persistent disagreements were settled by an experienced reviewer (AW). Articles deemed potentially relevant were extracted for full-text screening, following the same procedures as the abstract screening.

### Data extraction

The six reviewers (M.B., S.L., V.L., P.Y., Y.C and H.L.) independently extracted data from the included studies using a standardized form. To ensure accuracy and reliability, the extracted data were cross-checked by one of the six reviewers. The extracted data included authors, year of publication, country/region, demographics, population, study design, recruitment methods, diagnostic criteria for LBP and frailty, clinical outcomes related to LBP (e.g., pain intensity, presence of LBP, LBP-related disability in older adults) and frailty (frailty status), reported associations between these LBP-related measures and frailty, corresponding statistical analysis, and covariates defined by the authors. The primary outcomes of interest were the cross-sectional, temporal, or causal associations between LBP and frailty, expressed through various statistical measures, such as odds ratios (ORs), Pearson’s correlation coefficient, Spearman rho, relative risk, simple linear regression coefficient, or hazard ratio. The corresponding 95% confident intervals (95% CIs) were also noted, with or without adjustments for confounders. If both univariate and multivariate analyses were conducted in the original study, both unadjusted and adjusted association variables along with the corresponding 95% Cis, were extracted. If the original study did not clearly report statistical results, the corresponding authors were contacted for relevant information. Alternatively, association variables (e.g., ORs, Pearson correlation coefficients, or Spearman rho) and the corresponding 95% CIs were calculated from available raw data, if possible.

### Methodological quality assessments

Two reviewers (P.Y. & Y.C.) independently evaluated the methodological quality of the included studies using the Risk of Bias in Non-randomized Studies - of Exposure (ROBINS-E) tool [28] and the Appraisal tool for Cross-Sectional Studies (AXIS) tool [29] for longitudinal studies and cross-sectional studies, respectively. They resolved any disagreements through discussion, and if disagreements persisted, a third reviewer was consulted. The ROBINS-E tool assesses risk of bias across seven domains: (1) confounders; (2) the measurement of the exposure; (3) the selection of participants into the study (or into the analysis); (4) post-exposure interventions; (5) missing data; (6) the measurement of the outcome; and (7) the selection of the reported results [28]. Overall judgments regarding the risk of bias were classified as low, some concerns, high, and very high. The AXIS comprises 20 questions, and responses of ‘yes’, ‘no’, or ‘don’t know’ were recorded for each question to aid in grading the risk of bias of the included study [30].

### Certainty of evidence assessments

Two authors (P.Y. & Y.C.) independently assessed the certainty of evidence regarding the association between LBP intensity or LBP-related disability and frailty in older adults using the Adapted Grading of Recommendations Assessments, Development and Evaluation (GRADE) approach [31]. The GRADE framework is a transparent method for summarizing evidence and making clinical practice recommendations. Although it is commonly used for assessing evidence from randomized and non-randomized controlled trials, the principles of the GRADE approach have also been rigorously applied to evaluate the certainty of evidence in observational studies, including cross-sectional studies [32]. The certainty of evidence was assessed and categorized as high, moderate, low, or very low based on factors such as the risk of bias in the primary studies, inconsistency, imprecision, indirectness, and effect size [31]. Evidence from randomized trials start at a high level of certainty, whereas evidence from non-randomized studies—such as observational, cross-sectional, or case-control studies—start at a low level of certainty due to potential biases introduced by the absence of randomization, such as confounding and selection bias [33]. If the risk of bias of a given included study was moderate or high, the evidence would be downgraded. Evidence could be upgraded if most included studies reported a moderate to large similar effect, or if a potential exposure-gradient response was observed within and between the included studies [31].

### Statistical analysis

Meta-analyses were planned using Review Manager 5 (Version 5.4. Copenhagen: The Cochrane Collaboration, 2020) if two or more included studies reported the same association (e.g., cross-sectional or temporal), The I^2^ statistic was used to assess statistical heterogeneity, with I^2^ values classified as follows: might not be important (< 40%), moderate heterogeneity (30–60%), substantial heterogeneity (50–90%), or considerable heterogeneity (75–100%) [34]. Separate meta-analyses were planned for clinically and statistical homogeneous data, such as studies with the same design or statistical approach in examining associations at the same timepoint or data from individuals with the same LBP chronicity. However, due to significant clinical (e.g., different study designs, outcome measures, follow-up timepoints, definitions of frailty, and LBP chronicity) and statistical heterogeneity (e.g., multivariate logistic regression, multivariate linear regression, analysis of variance, generalized estimated equation) among the included studies, meta-analyses were not performed for the association between LBP-related measures and frailty. Instead, a narrative synthesis was used to summarize our findings. Specifically, studies reporting similar cross-sectional or temporal association between LBP-related outcomes (e.g., LBP status, chronicity, intensity, disability) and frailty status (using various criteria) at the same timepoints were grouped and summarized narratively. Quantitative estimates, such as regression coefficients, 95% confidence intervals (CI), were reported where available.

Subgroup analyses were also planned based on: (1) age categories (i.e., 60–64 years [youngest-old], 65–74 years [young-old], 75–84 years [middle-old], or 85 years or above [oldest-old]); (2) ethnicity; and (3) gender. The statistical significance level was set at 0.05.

## Results

### Study selection

**Fig. 1:**
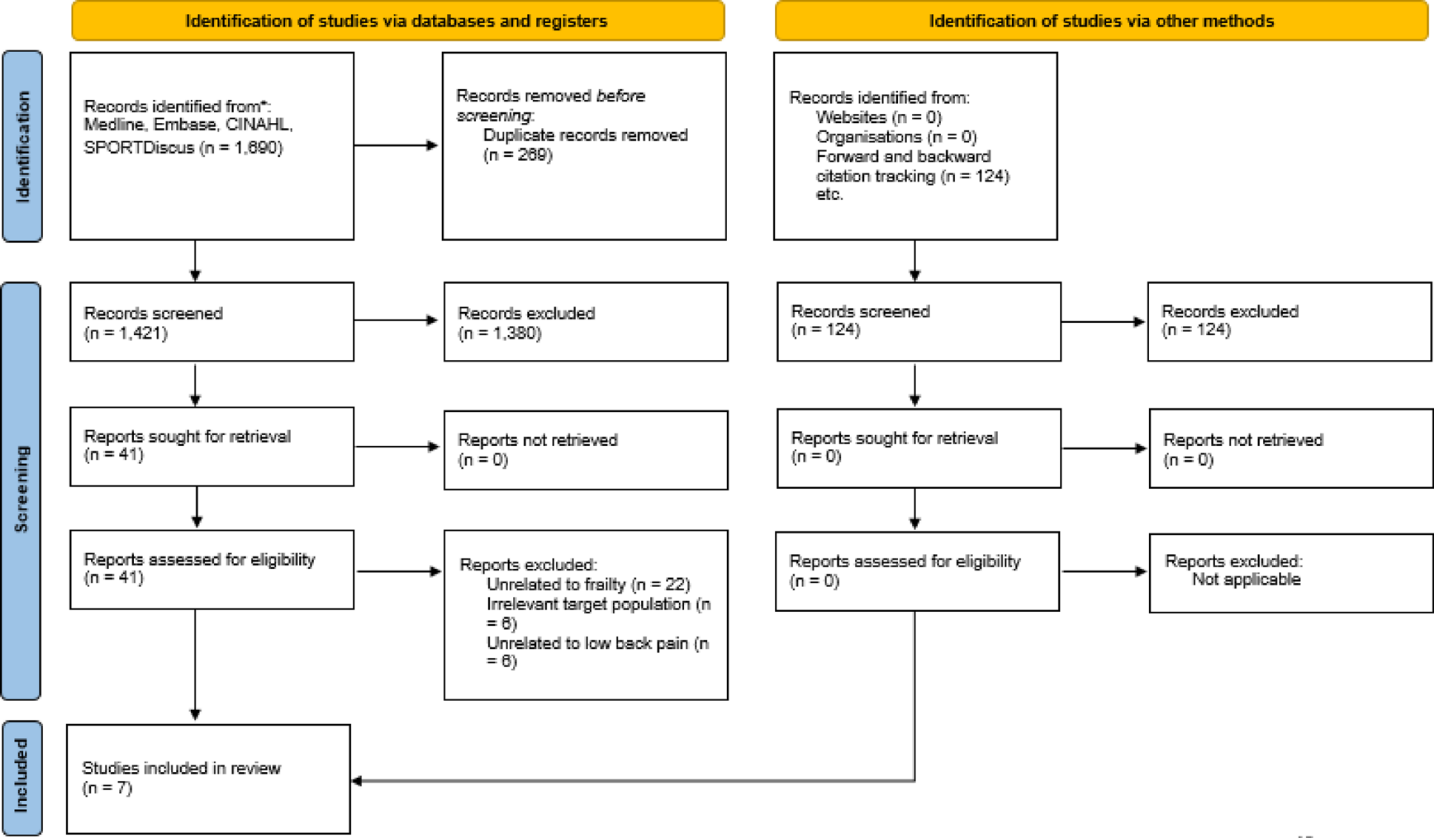
A flow diagram illustrating results from searches of databases and other sources

A total of 1,690 potential studies were identified from the database searches, and an additional 124 studies found through backward and forward citation tracking (Fig. 1). After removing duplicates and screening titles and abstracts, 41 full-text articles were retrieved for further evaluation. Ultimately, seven articles were included in this review [20, 23, 27, 35, 36]. The articles excluded during full-text screening were those unrelated to frailty (e.g., lacking correlation analysis, or focusing on sarcopenia or physical inactivity instead of frailty) (*n* = 22), irrelevant to the target population (*n* = 6) or investigating musculoskeletal pain rather than LBP (*n* = 6). The reviewers demonstrated a high level of agreement during the screening process. (K *≥* 0.75).

### Study characteristics

Six cross-sectional studies [20, 23, 27, 35, 36], and one longitudinal study [23], published between 2015 and 2024, were included (Table 1). A total of 6,078 participants were involved across studies in Brazil (two studies), China, Japan, the Republic of Korea, Thailand, and the United States. However, one included study did not report the number of females aged 60 years or older [36]. In the remaining six studies, approximately 70.5% of the 2,227 participants were female [20, 23, 27, 35], Participants were recruited from community or primary care settings. Three studies involved participants with chronic LBP [21, 27, 35], one involved individuals with frequent LBP [36], another focused on those with acute LBP [23], one examined individuals with acute non-specific LBP [23], and one included a mixed sample of both acute and chronic LBP [22] (Table 1). Except for the study that explicitly reported including individuals with acute non-specific LBP, it was unclear whether participants in other studies had non-specific LBP [23].

**Table 1:** Study characteristics

Studies	Study design	Countries	Recruitment criteria for participants with LBP	Number of participants (% females)	Types of LBP	LBP related measures	Frailty measure
Coyle et al [21]	Cross-sectional	United states	Participants (60–85 years old) with chronic LBP with pain ≥ 3/10 on pain intensity rating, ≥ 4 days per week, and ≥ 3 months. Participants (60–85 years old) without LBP were also included.	*n* = 123 (72.6%) LBP = 66 No LBP = 57	CLBP	Pain thermometer (vertically oriented 0–10 Likert scale)	CHS
Kim et al [35]	Cross-sectional	Republic of Korea	Participants (*≥* 60 years) with chronic LBP and complete medical records, but no definition of chronic LBP	*n* = 1,054 (67.9%) only recruited those with CLBP	CLBP	NRS	Frailty phenotype questionnaire
Leopoldino et al [20]	Cross-sectional	Brazil	Participants (*≥* 55 years) with acute LBP were that occurred within 6 weeks of the enrolment period and preceded by ≥ 6 months of pain-free period.	*n* = 589 (84.9%)	Acute non-specific LBP	NRS / RMDQ	CHS
Qing et al [36]	Cross-sectional	China	Participants ≥ 60 years with or without frequent LBP	*n* = 3,249 (unknown female proportion)	Frequent LBP	A question asking for painful location	35-item FI (dividing the sum of all measured deficits by the number of age-related impairments)
Rocha et al [23]	Longitudinal	Brazil	Participants (*≥* 65 years) with acute LBP. No LBP history over last 6 months, experiencing new episode of acute LBP that persisted for *≤* 6 weeks Follow up at 6 and 12 months	*n* = 155 (100%)	Acute LBP	NPS / RMDQ	CHS
Thonprasertvat et al [27]	Cross-sectional	Thailand	Participants aged ≥ 60 years with LBP or degenerative disease visiting an orthopedic outpatient department	*N* = 165 (73.9%) All of them had CLBP	CLBP	NRS / ODI (56.4% *≤* 20%; 43.6% >20%)	MFI-11 > 0.27 means frailty (non-frail, pre-frail, frail categories)
Tsuji et al [22]	Cross-sectional	Japan	Participants aged ≥ 65 years who underwent a comprehensive health examination between November 2016 and December 2018.	*n* = 730 (66.8%)	A mixed sample of acute and chronic LBP	ODI	J-CHS

CHS, Cardiovascular Health Study criteria; CLBP, Chronic low back pain; FI ,frailty index; J-CHS, Japanese version of the Cardiovascular Health Study criteria; LB , low back pain; MFI-11 , Modified frailty index 11; NPS , Numeric Pain Scale; NRS , Numeric Rating Scale; ODI, Oswestry Disability Index; ORs, odds ratios; RMDQ, Roland Morris Disability Questionnaire.

### Diagnostic methods for frailty

Three included studies [20, 21, 23] used the Frailty Phenotype described in CHS to assess frailty. Another included study [22] adopted the revised Japanese version of the Cardiovascular Health Study criteria (J-CHS), while another [35] used Frailty Phenotype Questionnaire (FPQ) to determine frailty. Both CHS and J-CHS evaluate frailty based on criteria such as shrinking (weight loss), weakness (grip strength), exhaustion, slowness (gait speed), and low activity level (Table 2). Similarly, the FPQ assesses frailty through weight loss, resistance (lower limb strength), fatigue, ambulation (walking distance), and inactivity. Individuals were categorized as frail if they met three or more criteria, pre-frail if they met one or two, and non-frail if they met none.

One included study used the modified frailty index-11 (mFI-11) [27], which evaluates the presence of 11 conditions or comorbidities (i.e., dependent functional status, history of diabetes mellitus, history of chronic obstructive pulmonary disease or pneumonia, history of congestive heart failure, myocardial infraction, history of percutaneous coronary intervention, hypertensive medications, peripheral vascular disease or rest pain, impaired sensorium, transient ischemic attack or cerebrovascular accident, and history of cerebrovascular accident with residual deficit). The overall score was calculated by dividing the number of conditions by 11 [27], with a score of 0.27 or higher indicates frailty [27]. Another study employed a 35-item Frailty Index to evaluate six potential domains of health deficits: activities of daily living (6 items), instrumental activities of daily living (5 items), physical functional limitations (9 items), chronic diseases (9 items); mental health (5 items), and self-rated health (1 item) [36]. The index score was obtained by dividing the sum of full or partial presence of health deficits by the total number of potential age-related impairments, with higher scores indicating greater frailty. However, this study only categorized frailty into four severity quarters without specifying a cutoff score to determine pre-frail or frail status.

**Table 2:** Items in cardiovascular health study (CHS) frailty scale, and frailty phenotype questionnaire

Item	CHS frailty scale	Item	Frailty phenotype questionnaire
Shrinking	Unintentionally loss of *≥* 2 kg in the past 6 months	Loss of weight	Unintentional weight loss of *≥* 4.5 kg in the past 12 months
Weakness	Grip strength < 28 kg in men, or < 18 kg in women	Resistance	Having difficulty in ascending 10 stairs by themselves without using an aids
Exhaustion	In the past 2 weeks, have you felt tired without any reason?	Fatigue	For the past week, I’ve felt like everything I do is an effort’. This feeling appeared > 3 days a week
Slowness	Gait speed < 1.0 m/s	Ambulation	Any level of difficulty (such as ‘a bit’, ‘very’, or ‘unable to do it at all’) in walking around a 400 m playground track
Low activity	Not engaging in moderate levels of physical exercise, or sports	Inactivity:	No moderate-intensity physical activity or vigorous physical activity at least once in the past 7 days Moderate-intensity physical activity included fast walking, carrying light objects, cleaning, and infant care; Vigorous physical activity included activities such as lifting or transporting heavy objects > 20 kg, digging, construction labouring, and carrying objects upstairs.

One point was assigned for the presence of a phenotype item in respective scale or questionnaire. Individuals with a score of 0 on each scale/questionnaire were categorized as no frailty, those with a score of 1 or 2 were classified as pre-frail, and those with a score of *≥* 3 were identified as frail

### Definitions of LBP

The definitions, assessment tools, and classification of LBP varied across the included studies. Although all studies recruited participants with LBP, the criteria differed. Two studies [20, 23] enrolled individuals with LBP lasting up to six weeks who had not received relevant treatment in the six months before the enrolment. One included study [21] involved participants with LBP lasting at least 3 months, while another study [22] used a single question - “Do you feel low back pain in your daily life lately?” - to determine the presence of LBP. Two included studies recruited older adults with chronic LBP (CLBP) without specifying further definitions [27, 35]. Another included study involved older adults with frequent LBP but did not provide a definition [36].

### LBP outcome measures

#### LBP intensity

For assessing LBP intensity, four included studies [20, 23, 27, 35] used the 11-point Numeric Pain Scale or NRS to determine LBP intensity, where 0 indicates no pain and 10 means the worst imaginable pain. Similarly, one study used a pain thermometer (vertically oriented 11-point Likert scale) to determine pain intensity [21]. Another study only used a single question to determine the painful location [36].

### LBP-related disability

Two included studies [20, 23] used the Roland Morris Disability Questionnaire (RMDQ) to measure LBP-related disability, while two other studies employed the Oswestry Disability Index [22, 27].

### Risk of bias assessments

The six included cross-sectional studies [20, 22, 27, 35, 36] displayed low (*n* = 3) and moderate (*n* = 3) risk of bias (Table 3). Common biases observed in these studies included a lack of strategies for addressing or categorizing non-responders and no detailed descriptions of non-responders’ characteristics, particularly concerning the response rate. The single prospective study [23] had a high risk of bias due to issues related to the handling of missing data (Table 4).

**Table 3:** Risk of bias assessments of the included cross-sectional studies

Studies	Objective and study design				Study participation						Handling of non-respondents				Outcome measures			Statistical analysis			Reporting							Overall risk
Original item number	Were the aims/objectives of the study clear?	Was the study design appropriate for the stated aim(s)?	**S**	Was the sample size justified?		Was the target/reference population clearly defined? (Is it clear who the research was about? )	Was the sample frame taken from an appropriate population base so that it closely represented the target/reference population under investigation?	Was the selection process likely to select subjects/participants that were representative of the target/reference population under investigation?	Was ethical approval or consent of participants attained?	**S**	Were measures undertaken to address and categorize non-responders?	Does the response rate raise concerns about non-response bias?	If appropriate, was information about non-responders described?	**S**	Were the risk factor and outcome variables measured appropriate to the aims of the study?	Were the risk factor and outcome variables measured correctly using instruments/ measurements that had been trialled, piloted or published previously?	**S**	Is it clear what was used to determined statistical significance and/or precision estimates? (e.g., p values, CIs)	Were the methods (including statistical methods) sufficiently described to enable them to be repeated?	**S**	Were the basic data adequately described?	Were the results internally consistent?	Were the results for the analyses described in the methods, presented? Discussion	Were the authors’ discussions and conclusions justified by the results?	Were the limitations of the study discussed?	Were there any funding sources or conflicts of interest that may affect the authors’ interpretation of the results?	**S**	
Coyle et al [21]	Y	Y	L	Y		Y	Y	Y	Y	L	N	N	N	H	Y	Y	L	Y	Y	L	Y	Y	Y	Y	Y	N	L	L
Kim et al [35]	Y	Y	L	N		Y	Y	U	Y	M	N	U	N	H	Y	Y	L	Y	Y	L	Y	Y	Y	Y	Y	N	L	M
Leopoldino et al [20]	Y	Y	L	Y		Y	Y	Y	Y	L	Y	N	N	H	Y	Y	L	Y	U	M	Y	Y	Y	Y	Y	N	L	M
Qing et al [36]	Y	Y	L	Y		Y	Y	Y	Y	L	N	U	N	H	Y	Y	L	Y	Y	L	Y	Y	Y	Y	Y	N	L	L
Thonprasertvat et al3 [27]	Y	Y	L	Y		Y	Y	Y	Y	L	N	N	N	H	Y	Y	L	Y	Y	L	Y	Y	Y	Y	Y	N	L	L
Tsuji et al [22]	Y	Y	L	N		Y	Y	U	Y	M	U	U	U	H	Y	Y	L	Y	Y	L	Y	Y	Y	Y	Y	N	L	M
% of studies that have “yes”/ no bias	100	100		60		100	100	60	100		20	0	0		100	100		100	80		100	100	100	100	100	0		

H, High risk of bias; L, low risk of bias; M, Moderate risk of bias; N,  No; S = Summary of a domain; U, Uncertain; Y,  Yes

**Table 4:** Risk of bias assessments of the included prospective longitudinal study

Study	Risk of bias due to confounding						Risk of bias arising from measurement of the exposure			Risk of bias in selection of participants into the study			Risk of bias due to post-exposure interventions			Risk of bias due to missing data								Risk of bias arising from measurement of the outcome				Risk of bias in selection of the reported result						Overall risk
Original Item Number	Did the authors control for all the important confounding factors for which this was necessary?	Were confounding factors that were controlled for (and for which control was necessary) measured validly and reliably by the variables available in this study?	Did the authors control for any variables after the start of the exposure period being studied that could have been affected by the exposure?	Did the use of negative controls, or other considerations, suggest serious uncontrolled confounding?	S	Does the measured exposure well-characterize the exposure metric specified to be of interest in this study?		Was the exposure likely to be measured with error, or misclassified?	S	Did follow-up begin at (or close to) the start of the exposure window for most participants?	Was selection of participants into the study (or into the analysis) based on participant characteristics observed after the start of the exposure window being studied?	S	Were there post-exposure interventions that were influenced by prior exposure during the follow-up period?	S	Were complete data on exposure status available for all, or nearly all, participants?		Were complete data on the outcome available for all, or nearly all, participants?	Were complete data on confounding variables available for all, or nearly all, participants?	Is the result based on a complete case analysis?	Was exclusion from the analysis because of missing data (in exposure, confounders or the outcome) likely to be related to the true value of the outcome?	Is there evidence that the result was not biased by missing data?	S	Could measurement or ascertainment of the outcome have differed between exposure groups or levels of exposure?		Were outcome assessors aware of study participants’ exposure history?	Could assessment of the outcome have been influenced by knowledge of participants’ exposure history?	S	Was the result reported in accordance with an available, pre-determined analysis plan?	Is the reported effect estimate likely to be selected, based on desirability of the magnitude (or statistical significance) of the estimated effect of exposure on outcome, from multiple exposure measurements within the exposure domain?	Is the reported effect estimate likely to be selected, based on desirability of the magnitude (or statistical significance) of the estimated effect of exposure on outcome, from multiple outcome measurements within the outcome domain?	Is the reported effect estimate likely to be selected, based on desirability of the magnitude (or statistical significance) of the estimated effect of exposure on outcome, from multiple analyses of the exposure-outcome relationship?	Is the reported effect estimate likely to be selected, based on the basis of desirability of the results (e.g. statistical significance), from different subgroups?	S	H
Rocha et al., 2023 [23]	Y	Y	N	N	L	Y		N	L	Y	N	L	N	L	Y		N	Y	Y	Y	N	H	N		Y	N	L	N	N	N	N	N	L	

H ,High risk of bias; L, Low risk of bias; M , Moderate risk of bias; N , No; S ,  Summary of a domain; Y, Yes

### Associations between LBP-related clinical outcomes and frailty status

According to the GRADE, the certainty of evidence across the included studies was determined to range from low to very low (Appendix II). Specifically, the certainty of evidence from the sole included prospective study [23] was rated as very low, primarily due to a high risk of bias from inadequate handling of missing data. The imprecision was also another reason for the downgrade (Table 5, Appendix II). Five key associations were identified: (1) cross-sectional associations between the presence of LBP and frailty status [21, 22, 36]; (2) cross-sectional associations between LBP intensity and frailty status [20, 21, 35]; (3) cross-sectional associations between LBP-related disability and frailty status [20, 22, 27]; (4) temporal association between baseline acute LBP intensity and future frailty status [23]; and (5) temporal association between baseline acute LBP intensity and future frailty status [23]. Four statistical tests were used to examine the cross-sectional associations between LBP intensity or LBP-related disability and frailty status: chi-square test [21], univariate logistic regression [22, 27, 36], multivariate logistic regression [22, 27], and multivariate linear regression [20]. Analysis of variance [35] was used to compare chronic LBP intensity between older adults with and without frailty in one study. Another study used generalized estimated equation [23] to determine whether greater baseline acute LBP or LBP-related disability predicted increased frailty progression in older females in 6 or 12 months.

### Cross-sectional associations between the presence of LBP and frailty status

Low-certainty evidence suggested a cross-sectional association between the presence of acute LBP, frequent LBP, or CLBP and frailty (Table 5) [21, 22, 36]. Coyle et al. (2015) found that individuals with CLBP were 7.5 times more likely to be prefrail or frail (UOR = 7.47, 95%CI: 3.35 to 16.63; *P* < 0.01) compared to asymptomatic controls [21] (Table 5). Tsuji et al. (2020) also reported that the presence of acute LBP or CLBP was associated with a prefrail or frail status (UOR = 1.71, 95% CI 1.52–9.05, *P* = 0.04; AOR = 3.41, 95% CI 1.39–8.39, *P* < 0.01) compared to asymptomatic controls (Table 5) [22]. Likewise, Qing et al., 2024 found that older adults with Frailty Index scores in the third or fourth quartile were more likely to have frequent LBP than those with scores in the 25th to 50th quartile, with UORs ranging from 1.34 to 2.69 [36].

### Cross-sectional associations between LBP intensity and frailty status

Two cross-sectional studies [21, 35] provided low-certainty evidence that chronic LBP intensity was associated with frailty when analyses were not adjusted for covariates (Table 5). Coyle et al., found that older adults with severe CLBP intensity (*≥* 5/10 on 11-point NRS) were 5.97 times more likely to be prefrail or frail than those with mild to moderate CLBP intensity (< 5/10 on NRS), without considering covariates (Table 5 [21]. Similarly, Kim et al. revealed that CLBP NRS scores were higher in prefrail or frail older adults, with the highest NRS scores observed in the frail group (*P* < 0.01) (Table 5) [35]. However, low-certainty evidence suggested that there was no significant association between acute LBP intensity and frailty after adjusting for clinical factors. Specifically, Leopoldino et al., found that the initially observed higher NRS scores of acute LBP in prefrail or frail older adults, compared to non-frail counterparts were no longer significant after adjusting for clinical variables (BMI, depressive symptoms, and co-morbidities) [20] (Table 5), both without and after accounting for sociodemographic variables (age, sex, marital status, education level, and income). Likewise, they found that frail older adults were expected to have NRS scores 1.07 to 1.15 points higher than non-frail counterparts, regardless of considering sociodemographic covariates [20] (Table 5), suggesting a positive association between acute LBP intensity and frailty [20] (Table 5). However, the same study showed that the differences in NRS scores between prefrail or frail older adults and non-frail older adults disappeared after adjusting for clinical variables (BMI, depressive symptoms, and co-morbidities) [20] (Table 5), suggesting.

**Table 5:** Cross-sectional and Temporal associations between low back pain-related clinical outcomes and frailty from included studies

Study	Dependent variables	Independent variables	Statistical tests used to determine the association	Statistics (e.g. odds ratio) (CI, 95%)	Association with frailty	Direction of the association	Certainty of evidence (Appendix II)
*Cross-sectional association between the presence of LBP and frailty*							
Coyle et al [21]	Presence of CLBP	Frailty (Robust vs. Pre-frail + frail) based on CHS	Chi-square test Odds ratio (calculated from raw data)	χ^2^ = 26.368, *p* < 0.001 UOR = 7.467 (3.353 to 16.628) *P* < 0.0001)	CLBP group had a greater proportion of pre-frail and frail older adults compared to the No CLBP group	Positive	Low
Qing et al [36]	Self-reported frequent LBP	Q1 FI quartile: *≤* 0.15 Q2 FI quartile: 0.16–0.18 Q3 FI quartile: 0.19–0.22 Q4 FI quartile: *≥* 0.23	Univariate logistic regression	Q2 FI reference group Q1 F1: UOR = 0.80 (0.67–1.2) Q3 FI: UOR = 1.34 (1.04–1.74) Q4 FI: UOR = 2.69 (2.11–3.41)	More severe frailty in Q3 and Q4 was related to the presence of frequent LBP	Positive	Low
Tsuji et al [22]	Frailty (Robust vs. prefrail + frail) based on J-CHS	Prevalence of LBP	Univariate and multivariate logistic regression	UOR = 1.61 (1.19–2.19), *p* = 0.002 AOR = 1.59 (1.17–2.16), *p* = 0.003 Adjusted for age, sex and BMI	Individuals with LBP were more likely to have prefrail or frail status	Positive	Low
	Frailty (Robust + prefrail vs. frail) based on J-CHS	Prevalence of LBP	Univariate and multivariate logistic regression	UOR = 3.71 (1.52–9.05), *p* = 0.004 AOR = 3.41 (1.39–8.39), *p* = 0.008 Adjusted for age, sex and BMI	Individuals with LBP were more likely to have frail status as compared to robust/ prefrail group	Positive	Low
*Cross-sectional Association between LBP intensity and frailty*							
Coyle et al [21]	CLBP intensity (categorical high (*≥* 5/10) vs. low (< 5/10 on pain thermometer)	Frailty (Pre-frail or frail) based on CHS	Chi-square test Odds ratio (calculated from raw data)	χ^2^ = 9.789, *p* = 0.0018 UOR = 5.971 (1.842 to 19.358) *P* = 0.0029)	High intensity CLBP group had a greater proportion of pre-frail or frail older adults compared to the low intensity CLBP group	Positive	Low
Kim et al [35]	CLBP intensity (NRS)	Robust, Prefrail, frail based on Frailty Phenotype questionnaire	Analysis of variance	Frail: 7.00 ± 1.83, Prefrail: 6.69 ± 1.71, Robust: 6.19 ± 1.90, *p* < 0.01	The NRS scores significantly increase with higher degree of frailty	Positive	Low
Leopoldino et al [20]	Acute non-specific LBP intensity (NRS)	Pre-frail (Robust vs. pre-frail) based on CHS	Linear regression (unadjusted) Multivariate linear regression (adjusted for sociodemographic variables) Multivariate linear regression (adjusted for clinical variables) Multivariate linear regression (adjusted for both sociodemographic and clinical variables)	Unadjusted β = 0.65 (CI, 0.12 to 1.17), *p* = 0.016 Adjusted β = 0.53 (CI, 0.01 to 1.04), *p* = 0.046 Adjusted β = 0.35 (CI, -0.23 to 0.94), *p* = 0.234 Adjusted β = 0.28 (CI, -0.30 to 0.86), *p* = 0.343	The unadjusted model showed that NRS scores of pre-frail older adults with LBP were expected to be 0.65 higher than non-frail individuals with LBP. The model adjusted sociodemographic variables (i.e., age, sex, marital status, education level, and income) showed that NRS scores of pre-frail older adults with LBP were expected to be 0.53 higher than non-frail individuals with LBP. The association between LBP intensity and frailty status became non-significant after adjusting for clinical variables (i.e., BMI, depressive symptoms, and co-morbidities) or both sociodemographic and clinical variables	Positive	Low
	Acute non-specific LBP intensity (NRS)	Frailty (Robust vs. Frail) based on CHS	Linear regression (unadjusted) Multivariate linear regression (adjusted for sociodemographic variables, i.e., age, sex, marital status, education level, and income)) Multivariate linear regression (adjusted for clinical variables) Multivariate linear regression (adjusted for both sociodemographic and clinical variables)	Unadjusted β = 1.15 (CI, 0.50 to 1.80), *p* = 0.001 Adjusted β = 1.07 (CI, 0.42 to 1.71), *p* = 0.001 Adjusted β = 0.68 (CI, -0.10 to 1.47), *p* = 0.086 Adjusted β = 0.65 (CI, -0.13 to 1.42), *p* = 0.103	The unadjusted model showed that NRS scores of frail older adults with LBP were expected to be 1.15 higher than non-frail individuals with LBP The model adjusted sociodemographic variables showed that NRS scores of frail older adults with LBP were expected to be 1.07 higher than non-frail individuals with LBP. The association between LBP intensity and frailty status became non-significant after adjusting for clinical variables (i.e., BMI, depressive symptoms, and co-morbidities) or both sociodemographic and clinical variables	Positive	Low
*Temporal association between baseline acute LBP intensity and future frailty status*							
Rocha et al [23]	Frailty (non-frail, prefrail and frail) based on CHS	Time: Baseline acute non-specific pain (NPS)	Generalized estimated equations Adjusted for baseline frailty	Adjusted β = -0.73, *p* = 0.001	Greater baseline acute LBP intensity was associated with increased progression of frailty in older females at the 6- and 12-month follow-up periods. The authors suggested that the progression stabilized or even showed some improvement by the 12-month follow-up.	Positive	Very low
*Cross-sectional association between LBP-related disability and frailty*							
Leopoldino et al [20]	Disability (RMDQ) related to acute non-specific LBP	Frailty (Robust vs. pre-frail) based on CHS	Linear regression (unadjusted) Multivariate linear regression (adjusted for sociodemographic variables) Multivariate linear regression (adjusted for clinical variables) Multivariate linear regression (adjusted for both sociodemographic and clinical variables)	Unadjusted β = 3.83 (CI, 2.70 to 4.95), *p* < 0.001 Adjusted β = 3.51 (CI, 2.39 to 4.62), *p* < 0.001 Adjusted β = 1.84 (CI, 0.72 to 2.96), *p* = 0.001 Adjusted β = 1.68 (CI, 0.56 to 2.80), *p* = 0.003	The unadjusted model showed that RMDQ scores of pre-frail older adults with LBP were expected to be 3.83 points higher than non-frail individuals with LBP. The model adjusted for sociodemographic variables (i.e., age, sex, marital status, education level, and income) showed that RMDQ scores of pre-frail older adults with LBP were expected to be 3.51 points higher than non-frail individuals with LBP. The model adjusted for clinical variables (i.e., BMI, depressive symptoms, and co-morbidities) showed that RMDQ scores of pre-frail older adults with LBP were expected to be 1.84 points higher than non-frail individuals with LBP. The model adjusted for both sociodemographic and clinical variables showed that RMDQ scores of pre-frail older adults with LBP were expected to be 1.68 points higher than non-frail individuals with LBP.	Positive	Low
	Disability (RMDQ) related to acute non-specific LBP	Frailty (Robust vs. frail) based on CHS	Linear regression (unadjusted) Multivariate linear regression (adjusted for sociodemographic variables) Multivariate linear regression (adjusted for clinical variables) Multivariate linear regression (adjusted for both sociodemographic and clinical variables)	Unadjusted β = 7.24 (CI, 5.84 to 8.63), *p* < 0.001 Adjusted β = 6.72 (CI, 5.31 to 8.13), *p* < 0.001 Adjusted β = 3.99 (CI, 2.50 to 5.49), *p* < 0.001 Adjusted β = 3.69 (CI, 2.19 to 5.19), *p* < 0.001	The unadjusted model showed that RMDQ scores of frail older adults with LBP were expected to be 7.24 points higher than non-frail individuals with LBP. The model adjusted for sociodemographic variables (i.e., age, sex, marital status, education level, and income) showed that RMDQ scores of frail older adults with LBP were expected to be 6.72 points higher than non-frail individuals with LBP. The model adjusted for clinical variables (i.e., BMI, depressive symptoms, and co-morbidities) showed that RMDQ scores of frail older adults with LBP were expected to be 3.99 points higher than non-frail individuals with LBP. The model adjusted for both sociodemographic and clinical variables showed that RMDQ scores of frail older adults with LBP were expected to be 3.69 points higher than non-frail individuals with LBP.	Positive	Low
Thonprasertvat et al [27]	Disability (ODI-Thai version)	Frailty (score by MFI-11)	Univariate and multivariate logistic regression	UOR = 1.787, (CI, 1.261 to 2.533), *p* = 0.001 AOR = 1.740 (CI, 1.146 to 2.641), *p* = 0.009 Adjusted for age, gender, pain score,	A one-point increase in the Frailty score resulted in a 79% higher likelihood of experiencing severe disability (ODI score between 41% and 60%) in the crude regression model, and a 74% higher likelihood in the adjusted regression model.	Positive	Low
Tsuji et al [22]	Frailty (Robust vs. prefrail + frail) Based on J-CHS	Disability (ODI)	Univariate and multivariate logistic regression	UOR = 1.05 (CI, 1.04–1.07), *p* < 0.001 AOR = 1.05 (CI, 1.03–1.07), *p* < 0.001 Adjusted for age, gender (female), BMI	The ODI scores were associated with a status of prefrail/frail group in both crude and adjusted models.	Positive	Low
	Frailty (Robust + prefrail vs. frail) Based on J-CHS	Prevalence of LBP	Univariate and multivariate logistic regression	UOR = 1.07 (CI, 1.05–1.10), *p* < 0.001 AOR = 1.06 (CI, 1.04–1.09), *p* < 0.001 Adjusted for age, gender (female), BMI	The ODI scores were associated with the frail group in both crude and adjusted models.	Positive	Low
*Temporal association between baseline RMDQ scores and future frailty status*							
Rocha et al [23]	Frailty based on CHS	Time (6- and 12-month follow-up periods): acute baseline LBP-related disability (RMDQ)	Generalized estimated equations	Adjusted β = -0.74, *p* = 0.001	Higher baseline RMDQ scores was associated with increased progression of frailty in older females at the 6- and 12-month follow-up periods. The authors suggested that the progression stabilized or even showed some improvement by the 12-month follow-up.	Positive	Very low

AOR, adjusted odds ratio; CLBP, chronic low back pain; OR, odds ratio; RMDQ, Roland Morris Disability Questionnaire; CHS, Cardiovascular Health Study criteria; FI, frailty index; J-CHS, Japanese version of the Cardiovascular Health Study criteria; LBP, low back pain; MFI-11, Modified frailty index 11; NPS, Numerical Pain Scale; NRS, Numeric Rating Scale; ODI, Oswestry Disability Index; OR, odds ratio; β, regression coefficient; EQ-5D-5 L,  quality of life measure; SF-36, quality of life measure (36-item short form survey); UOR, unadjusted odds ratio

### Cross-sectional associations between LBP-related disability and frailty status

Three cross-sectional studies [20, 22, 27] provided low certainty of evidence indicating an association between acute or chronic LBP-related disability and frailty. Leopoldino et al. [20] revealed that the RMDQ scores of pre-frail older adults with acute non-specific LBP were 1.68 to 3.83 points higher than those of non-frail counterparts, depending on whether sociodemographic and clinical variables were adjusted. Similarly, frail older adults with acute non-specific LBP had RMDQ scores 3.69 to 7.24 points higher than non-frail older adults, depending on adjustments for sociodemographic and clinical variables (Table 5) [20]. Thomprasertvat et al. [27] found that each one-point increase in the mFI-11 score was associated with a 74–79% higher likelihood of experiencing severe disability (ODI scores between 41% and 60%) (Table 5). Tsuji et al. [22] reported that higher ODI scores were associated with prefrail and frail status in older adults, independent of age, gender, and BMI (Table 5).

### Temporal association between baseline acute LBP intensity or LBP-related disability and future frailty status

A longitudinal study by Rocha et al. [23] provided very low-certainty evidence that older women with higher acute LBP intensity (β = − 0.735) or LBP-related disability (β = − 0.745) at baseline experienced a worsened transition in their frailty status (from non-frail to prefrail or frail) over the subsequent 6 and 12 months. The study reported that 28.5% of non-frail older women at baseline became frail by the 6-month follow-up, all of whom had acute LBP at baseline. Rocha et al. suggested that the intensity of pain and the associated disability contributed to the worsening frailty status within 6 months, which then stabilized or improved once the pain subsided or stabilized [23].

## Discussion

This is the first systematic review to summarize evidence on the association between LBP-related measures and frailty among older adults. We found that a higher prevalence of LBP, increased chronic LBP intensity, and greater acute or chronic LBP-related disability were associated with frailty in older adults with LBP. However, the certainty of evidence for these cross-sectional associations was low. Notably, most of the identified associations were cross-sectional. Only one prospective study, which had a high risk of bias, reported that older women with higher acute LBP intensity or associated disability at baseline were likely to transition from no frailty to pre-frailty, or from pre-frailty to frailty over a 12-month period [23]. Despite these significant findings, the certainty of evidence regarding the temporal association between LBP intensity and frailty, or between LBP-related disability and frailty, was very low.

### Association of LBP prevalence and LBP intensity with frailty

Our review indicated that the presence of LBP or higher LBP intensity is associated with frailty in older adults. Older adults are known to have decreased homeostatic reserves to restore equilibrium when facing internal or external challenges, affecting their ability to handle biological, psychological, or social stressors. Chronic pain is a stressor that disrupts the homeostasis of older adults and may induce the onset or progression of frailty through multiple mechanisms such as reduced mobility, depression, kinesiophobia, social isolation, reduced nutritional intake, comorbidities, and polypharmacy [10, 37, 38]. These changes increase the vulnerability of older adults, compromising their ability to effectively manage various stressors [39], leading to the development of frailty. Additionally, chronic pain involves chronic low-grade inflammation. Pro-inflammatory cytokines (e.g., interleukin-6, and tumor necrosis factor-α) may trigger nociceptors, increase pain sensitivity, and lower pain threshold [40], while also causing muscle catabolism, resulting in sarcopenia in older adults [41]. The causal relationship between LBP and frailty status remains uncertain. A longitudinal study included in the current review found that older women with higher acute LBP intensity were more likely to progress through the stages of frailty over the ensuing 12 months. Prior prospective research has also shown that chronic widespread pain at baseline is associated with the development of frailty 4.3 years later [42]. A systematic review and meta-analysis of five prospective studies involving 13,120 older adults concluded that individuals with chronic pain at baseline had doubled the risk of developing frailty after 3 to 8 years [12]. However, that review was limited by heterogeneous assessments of pain and frailty in the primary studies. The type, duration, location, intensity, and etiology of pain in those studies were not clearly specified. As none of the included studies focused solely on CLBP, their findings cannot be directly compared with the current review. A recent prospective study published after our search period revealed that older adults with LBP had a reduced risk of prefrailty (AOR = 0.98) and did not significantly increase the risk of frailty over an eight-year period, after adjusting for various covariates [43]. Further prospective research is warranted to ascertain the causal relationship exists between acute or chronic LBP and frailty. Future trials should also investigate whether LBP interventions targeting older adults can reduce pain, enhance physical performance and prevent frailty in this population.

### Association between LBP-related disability and frailty

Three included studies found a positive association between LBP-related disability and frailty [20, 22, 27]. Given the variations in LBP-related disability questionnaires, LBP chronicity, and frailty scales in these studies, their results could not be pooled for a meta-analysis. However, the findings suggest a positive relation between LBP-related disability and frailty. LBP is a leading cause of disability worldwide [44, 45], commonly assessed using ODI and RMDQ. These questionnaires estimate impairments in muscle strength, activities of daily living, walking ability, physical activity, and social participation - key elements that also characterize frailty. A systematic review reported that individuals with CLBP and high disability levels exhibited low physical activity [46]. This low level of physical activity in older adults with CLBP increases their risk of developing disability, frailty, and persistent pain in a vicious cycle. The well-established association between activities of daily living, disability, and frailty [47] suggests that LBP-related disability may significantly contribute to frailty. Therefore, addressing LBP-related disability is crucial for improving frailty in older adults.

## Future research and clinical implications

In recent decades, there has been an increasing trend of middle-aged and older adults developing chronic pain [48]. Potential mechanisms linking aging and chronic pain include altered pain transmission and reduced pain sensitivity [48, 49]. One included study [21] suggested that decreased physical activity among older adults with LBP might contribute to the development of frailty, but more prospective studies are warranted to clarify the causal relation between CLBP and frailty. The associations identified from the included studies demonstrated very low- to low-certainty evidence because both cross-sectional and prospective studies are not considered high-quality designs compared to randomized controlled trials in the GRADE evaluation. To prevent downgrading due to high risk of bias the included studies, it is essential to address common sources of bias, such as inadequate handling of missing data and non-respondents. Importantly, minimizing attrition requires reducing participant burden, maintaining regular and meaningful engagement, providing incentives, and building strong rapport with participants. This can be achieved by paying attention to participants’ concerns and offering clear channels of communication with investigators, thereby fostering a sense of value, belonging, and commitment among participants [50]. Additionally, future randomized controlled trials should investigate whether effective and timely management of LBP can prevent the development or progression of frailty among older adults with LBP. Alternatively, clinical trials could evaluate the effects of frailty treatments (e.g., strengthening exercises, nutritional supplementation, and lifestyle modification) on physical resilience and the prevention or alleviation of LBP in older adults. Additionally, research should explore the correlation between the quality and quantity of paraspinal muscles and LBP intensity or disability in older individuals with or without frailty.

### Strengths of the current review

Determining the optimal dosage of multicomponent exercise programs for improving frailty status in older adults with varying severity of LBP and frailty is essential. Given the close association between CLBP and kinesiophobia, cognitive behavioural treatment [51] and cognitive function therapy [52] have shown significant benefits in reducing pain, kinesiophobia, and improving physical function in working-age adults. Systematic reviews have validated the effectiveness of spinal manipulative therapy (SMT) for treating LBP in older adults [53, 54]. An individual participant data meta-analysis reveals moderate certainty evidence that SMT, encompassing both manipulation and mobilization, offers comparable benefits for pain and function to older adults as interventions (mainly exercise therapy), which are recommended in international clinical practice guidelines [55]. These benefits persist in short-term (12 weeks), intermediate (26 weeks), and long-term (12 months). However, adverse reactions were often underreported, especially in older patients. The reported adverse events were likely to be more serious, or unrelated to SMT [56]. Although major adverse events are rare, clinicians should screen for contraindications and red flags, tailoring interventions for older adults with LBP as necessary. Future studies should investigate the effectiveness and safety of these interventions in improving LBP, disability, and frailty among older adults.

## Limitations

This review has several strengths. First, our protocol was registered with PROSPERO in advance, ensuring transparency. Second, multiple databases were searched to ensure a comprehensiveness in our findings. Additionally, standardized screening, data extraction, and risk of bias assessments were implemented to maintain methodological rigor. Third, the GRADE approach for cohort and cross-sectional studies was used to evaluate the certainty of evidence regarding the associations. This review had several limitations. First, only seven relevant studies were identified, which may affect the generalizability of the findings. Second, the certainty of evidence was low, as three included cross-sectional studies and one prospective study had moderate and high risk of bias, respectively. Third, as only one prospective study with a high risk of bias has been identified so far, future large-scale, high-quality prospective studies with long follow-ups are needed to explore the causal relationship between LBP and frailty. Additionally, researchers should consider conducting mediation analyses to explore other potential mediators (e.g., depression, physical activity levels, and social support) that may be modifiable. Fourth, due to the heterogeneity of the included studies, no meta-analysis was performed to quantify the associations between LBP-related measures and frailty. This limitation restricted our ability to determine the overall effect size of the associations. Therefore, only the patterns of association were summarized in this review.

## Conclusions

This review indicates that chronic LBP, acute or chronic LBP-associated disability, are significantly related to frailty status in older adults, although the certainty of evidence is low. Community-dwelling older adults with acute LBP, particularly those experiencing higher pain intensity, are more likely to develop frailty over time. However, due to the limited number of relevant studies, especially prospective ones, the current review only identifies patterns of association. Future large-scale, prospective studies should determine the causal relationship between different types or chronicity of LBP and frailty. Additionally, as the mechanisms underlying these associations remain unclear, future studies should explore potential mediators and moderators (e.g., depression, physical inactivity, fear avoidance behaviors) that may influence the association between LBP and frailty in older adults. Understanding these factors is crucial for developing more effective prevention and rehabilitation strategies to mitigate the impacts of LBP on frailty, and vice versa.

## Data Availability

Data is provided within the manuscript or supplementary information files.
